# Digital pathology and multimodal learning on oncology data

**DOI:** 10.1093/bjrai/ubae014

**Published:** 2024-09-11

**Authors:** Asim Waqas, Javeria Naveed, Warda Shahnawaz, Shoaib Asghar, Marilyn M Bui, Ghulam Rasool

**Affiliations:** Department of Cancer Epidemiology, H. Lee Moffitt Cancer Center & Research Institsute, Tampa, FL, 33612, United States; Machine Learning Department, H. Lee Moffitt Cancer Center & Research Institute, Tampa, FL, 33612, United States; Rashid Latif Medical College, Lahore, 54600, Pakistan; Mobile Infirmary Medical Center, Mobile, AL, 36607, United States; Rashid Latif Medical College, Lahore, 54600, Pakistan; Machine Learning Department, H. Lee Moffitt Cancer Center & Research Institute, Tampa, FL, 33612, United States; Department of Pathology, H. Lee Moffitt Cancer Center & Research Institute, Tampa, FL, 33612, United States; Morsani College of Medicine, University of South Florida, Tampa, FL, 33612, United States; Machine Learning Department, H. Lee Moffitt Cancer Center & Research Institute, Tampa, FL, 33612, United States; Morsani College of Medicine, University of South Florida, Tampa, FL, 33612, United States; Department of Neuro-Oncology, H. Lee Moffitt Cancer Center & Research Institute, Tampa, FL, 33612, United States

**Keywords:** artificial intelligence, machine learning, digital pathology, computational pathology, multimodal learning, oncology data integration, cancer research

## Abstract

Cancer presents a complex tapestry of biological, clinical, and molecular characteristics that collectively influence its diagnosis, progression, and treatment. This review article delves into the recent advancements in integrating multimodal oncology data, a pivotal aspect in the evolving field of digital pathology (DP). The complexity and heterogeneity of cancer, characterized by its multimodal nature, present significant challenges in diagnosis and treatment. Traditional methods of oncology data analysis often fall short of capturing the comprehensive landscape of cancer’s multifaceted characteristics. The advent of artificial intelligence, machine learning, and deep learning has marked a new era in multimodal learning. These technologies have revolutionized how oncologists and researchers understand and approach cancer, allowing for a more nuanced and detailed analysis. In this review article, we attempt to examine and present how DP enriches its methods and analysis with other data modalities, including clinical, radiological, and molecular information. We present opportunities and challenges of multimodal learning in oncology, highlighting the synergistic potential of combining various data types for improving cancer care with a focus on DP. Continuous innovation in multimodal data integration will be instrumental in transforming cancer diagnosis, treatment planning, prognosis, and post-treatment surveillance.

## Introduction

The examination of tissue samples under a microscope by pathologists is the standard procedure for disease diagnosis in clinical settings. However, technological advancements in the last couple of decades have ushered in a new era in the field of pathology called digital pathology (DP). Some of the key factors that have enabled the transformation from traditional pathology to DP include: (1) Whole Slide Imaging (WSI), which scans entire tissue sections on glass slides at high resolution and stores them as digital slides that can be analysed computationally,^1^ (2) Image analysis algorithms from the fields of computer vision and machine learning (ML) that can detect, segment, classify, and quantify morphological features, cells, tissues, biomarkers from digitized images,^2^ (3) Advanced imaging techniques such as computational staining and multiplexed imaging,^3^ (4) Molecular assays that generate quantitative molecular data from tissues using techniques such as polymerase chain reaction, microarrays, and next-generation sequencing,^4^ and (5) Enterprise imaging that includes pathology informatics.^5^

As the field of pathology evolves, so too does our understanding of cancer. Cancer is a complex disease that can manifest in different forms, each with its distinct characteristics and implications for screening, diagnosis, prognosis, and treatment. Over time, our understanding of cancer has evolved. Initially thought to be primarily a result of genetic mutations, it is now recognized as a complex interplay of genetic, environmental, and lifestyle factors. Advances in genomics, proteomics, bioinformatics, and imaging have led to a deeper understanding of cancer, revealing that even cancers originating in the same organ can have vastly different characteristics and responses to treatment. This complexity underscores the necessity for personalized medicine approaches tailored to the unique genetic makeup of each cancer and each patient.

Transitioning from the complexity of cancer biology to the use of advanced technologies, the success of artificial intelligence (AI) and ML in different domains of science, especially those focused on quantitative imaging analysis, has attracted anatomical pathology researchers and practitioners. As a result, computational pathology (CPATH) emerged when AI and ML models were introduced for various types of quantitative analysis in DP.^6^ CPATH uses AI and ML models to extract information from digital images that may extend beyond immediate human capabilities. By offering AI/ML-powered analytical tools, CPATH has the potential to enhance the roles of pathologists in clinical settings by making their work more efficient, accurate, and reproducible. This enhancement spans various aspects, including improving the precision, reproducibility, and scalability of tasks related to disease diagnosis and the quantification of biomarkers for prognosis and prediction.

However, while the integration of AI and ML into DP clinical workflow promises significant advances, it also presents several challenges. These challenges include a shortage of staff trained in both AI and DP that may temporarily lead to increased workloads primarily due to the learning curve in adoption of AI in the DP workflows. Nevertheless, over time, the increased use of AI and its incorporation into the training curriculum will gradually improve the number of AI-qualified individuals (see definition in Box 1) in the DP/CPATH field. The growing complexity of diagnostics due to evolving protocols and new biomarkers, as well as variations in rare diseases and morphological similarities, also pose challenges. Additionally, quality issues arising from tissue artefacts and staining/imaging inconsistencies, along with a lack of standardized practices and protocols, may impede interoperability and pose reproducibility challenges.

**Box 1.** Key pathology terminologies.

Pathology	Stems from the Greek word pathologia, meaning the study of suffering, it is a medical specialty investigating the origin, progression, and alterations in the structure, function, and natural course of diseases.^7^ There are two main branches of pathology: anatomical pathology (AP) focuses on studying tissue samples to diagnose diseases such as cancer, and clinical pathology (CP) analyses bodily fluids and conducts laboratory tests.^8^ Molecular pathology is an emerging branch that studies diseases at the molecular level by examining tissue or body fluids.
Histology	Microscopic examination of tissues and organs by sectioning, applying stains, and observing the prepared sections under a microscope. This process enables the visualization of tissue structures and any distinctive alterations the tissue might have undergone. Haematoxylin (a basic dye that stains cell nuclei in a purplish blue colour) and Eosin (an acidic dye that stains cytoplasm in a pinkish red colour), referred to as H&E, are commonly used together to stain structures of the cells to define intracellular organelles and proteins.^9^
Histopathology	It extends the utility of histological analysis by focusing on the study of disease indicators through the microscopic examination of processed and fixed glass slides containing sections from biopsies or surgical specimens. To visualize various tissue components under a microscope, these specimens are dyed with one or more stains depending on the investigational question being asked.^9^^,^^10^
Immunohistochemistry (IHC)	A valuable tool extensively employed in pathology for cell classification and diagnosis. Commonly performed on formalin-fixed paraffin-embedded (FFPE) tissue, IHC targets specific antigens in tissues and cells using antibodies to determine cell type and organ of origin. IHC usage has lately expanded to study predictive and prognostic biomarkers in oncological settings, making it a useful technique in modern pathology practice.^11^
WSI	The process of digitizing glass slides at multiple magnifications and focal levels to create digital images for observation and image analysis. WSI aims to replicate the experience of traditional light microscopy through digital means and is often known as virtual microscopy.^12^
Cytology slides	These refer to glass slides that contain specimens of individual cells or cell clusters obtained from various bodily fluids, tissues, or fine needle aspiration (FNA) biopsy. Unlike histology, which focuses on the function of tissues, cytology slides are prepared for microscopic examination to analyse cellular morphology, structure, and characteristics.^13^
Digital pathology (DP)	DP includes converting histopathology, IHC, or cytology slides into digital formats through whole-slide scanners, followed by applying computer-aided analytical tools for objective analysis. DP has improved pathology diagnoses through multiple techniques as discussed in this review.
CPATH	CPATH is the sub-branch of DP that involves computational analysis of digital slides. CPATH aims to analyse patient specimens by extracting information from digitized pathology images in combination with their associated meta-data, typically using AI methods to gain valuable insights into disease processes.^6^^,^^8^^,^^14^
Radiomics	Radiomics is a sub-field of radiology, where radiological images are converted into high-dimensional mine-able data for AI-based analysis to uncover biomarkers for diagnosis, prognosis, and treatment response prediction.^15^
Pathomics	Pathomics is a sub-field of pathology, that covers a wide variety of data captured from image analyses in the form of features that describe multiple phenotypic features of tissues in WSIs. Using AI, pathomics provides a quantitative assessment of identified structures and features to complement traditional histopathologic evaluation by pathologists.^16^
AI-qualified individuals	In the DP/CPATH context, individuals who not only understand AI at a basic technical level but also have the clinical, regulatory, and practical knowledge to apply AI effectively in pathology. This includes clinical validation, understanding regulatory and ethical implications, and the ability to implement, use, and interpret AI tools and their outcomes in daily practice. While it is optional but valuable that these individuals may also be involved in developing new AI tools, the term more broadly encompasses those who are well-equipped to work with AI in a clinical environment, ensuring its accurate, safe, and effective use.

**Box 1.** Key pathology terminologies.

Pathology	Stems from the Greek word pathologia, meaning the study of suffering, it is a medical specialty investigating the origin, progression, and alterations in the structure, function, and natural course of diseases.^7^ There are two main branches of pathology: anatomical pathology (AP) focuses on studying tissue samples to diagnose diseases such as cancer, and clinical pathology (CP) analyses bodily fluids and conducts laboratory tests.^8^ Molecular pathology is an emerging branch that studies diseases at the molecular level by examining tissue or body fluids.
Histology	Microscopic examination of tissues and organs by sectioning, applying stains, and observing the prepared sections under a microscope. This process enables the visualization of tissue structures and any distinctive alterations the tissue might have undergone. Haematoxylin (a basic dye that stains cell nuclei in a purplish blue colour) and Eosin (an acidic dye that stains cytoplasm in a pinkish red colour), referred to as H&E, are commonly used together to stain structures of the cells to define intracellular organelles and proteins.^9^
Histopathology	It extends the utility of histological analysis by focusing on the study of disease indicators through the microscopic examination of processed and fixed glass slides containing sections from biopsies or surgical specimens. To visualize various tissue components under a microscope, these specimens are dyed with one or more stains depending on the investigational question being asked.^9^^,^^10^
Immunohistochemistry (IHC)	A valuable tool extensively employed in pathology for cell classification and diagnosis. Commonly performed on formalin-fixed paraffin-embedded (FFPE) tissue, IHC targets specific antigens in tissues and cells using antibodies to determine cell type and organ of origin. IHC usage has lately expanded to study predictive and prognostic biomarkers in oncological settings, making it a useful technique in modern pathology practice.^11^
WSI	The process of digitizing glass slides at multiple magnifications and focal levels to create digital images for observation and image analysis. WSI aims to replicate the experience of traditional light microscopy through digital means and is often known as virtual microscopy.^12^
Cytology slides	These refer to glass slides that contain specimens of individual cells or cell clusters obtained from various bodily fluids, tissues, or fine needle aspiration (FNA) biopsy. Unlike histology, which focuses on the function of tissues, cytology slides are prepared for microscopic examination to analyse cellular morphology, structure, and characteristics.^13^
Digital pathology (DP)	DP includes converting histopathology, IHC, or cytology slides into digital formats through whole-slide scanners, followed by applying computer-aided analytical tools for objective analysis. DP has improved pathology diagnoses through multiple techniques as discussed in this review.
CPATH	CPATH is the sub-branch of DP that involves computational analysis of digital slides. CPATH aims to analyse patient specimens by extracting information from digitized pathology images in combination with their associated meta-data, typically using AI methods to gain valuable insights into disease processes.^6^^,^^8^^,^^14^
Radiomics	Radiomics is a sub-field of radiology, where radiological images are converted into high-dimensional mine-able data for AI-based analysis to uncover biomarkers for diagnosis, prognosis, and treatment response prediction.^15^
Pathomics	Pathomics is a sub-field of pathology, that covers a wide variety of data captured from image analyses in the form of features that describe multiple phenotypic features of tissues in WSIs. Using AI, pathomics provides a quantitative assessment of identified structures and features to complement traditional histopathologic evaluation by pathologists.^16^
AI-qualified individuals	In the DP/CPATH context, individuals who not only understand AI at a basic technical level but also have the clinical, regulatory, and practical knowledge to apply AI effectively in pathology. This includes clinical validation, understanding regulatory and ethical implications, and the ability to implement, use, and interpret AI tools and their outcomes in daily practice. While it is optional but valuable that these individuals may also be involved in developing new AI tools, the term more broadly encompasses those who are well-equipped to work with AI in a clinical environment, ensuring its accurate, safe, and effective use.

Amid these challenges, multimodal learning in oncology represents a cutting-edge approach to cancer research and treatment, leveraging the integration of various data types to gain a comprehensive understanding of cancer’s complexities.^17–19^ This approach combines diverse datasets, including genomic, proteomic, imaging, clinical, and demographic data, to develop a holistic view of cancer biology. By harnessing advanced computational techniques offered by AI/ML, multimodal learning enables analysing these disparate data types jointly in a unified framework.^8^^,^^19^ This integration is paramount for identifying novel biomarkers, elucidating tumour heterogeneity, predicting treatment responses, and ultimately facilitating the development of more effective, personalized therapies.^20^ Building upon these developments, DP and CPATH have paved the way for exploring novel methods for cancer diagnosis, prognosis, treatment planning, and also for research into a deeper understanding of cancer biology.^21–24^ The innovative algorithms within CPATH, especially those employing differential privacy and federated learning, enable the development of robust analytical models. These models are crucial for safeguarding patient privacy, fostering collaboration among institutions, and paving the way for the adoption of clinical-grade systems.^25^ Such advancements underscore the critical role of AI and ML in transforming cancer care, making it more precise, personalized, and effective.

## From conventional to digital pathology

**Figure 1.**
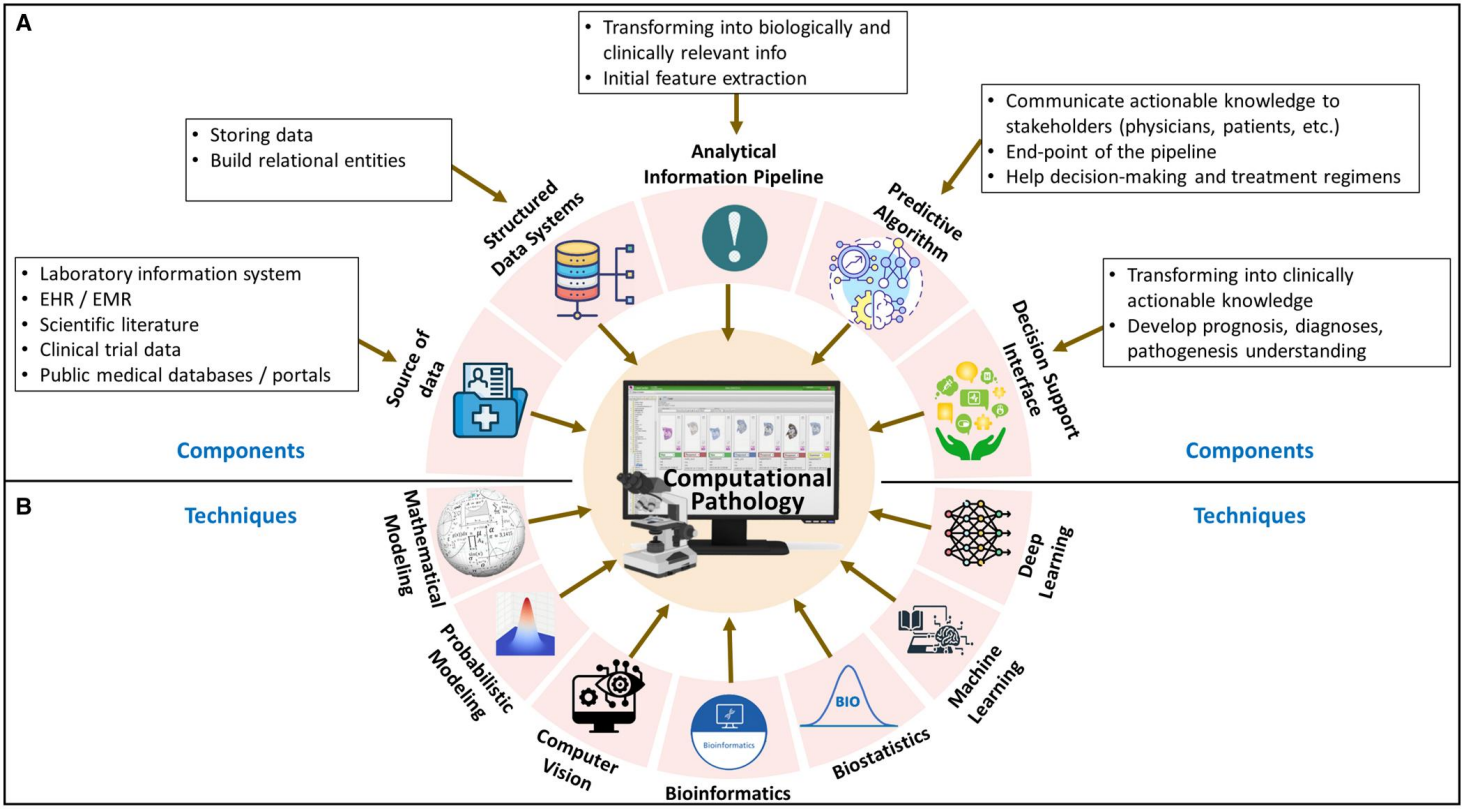
A template of the future road map for Digital Pathology (DP) and CPATH is presented. (A) Components of DP and CPATH are depicted, which include sources of input data, structured database systems, analytical information extraction pipeline, predictive algorithms, and decision support end-point interfaces. (B) Existing DP and CPATH techniques frequently used in research include mathematical models, probabilistic modelling, computer vision, bioinformatics, biostatistics, ML, and deep learning.

Conventionally, pathologists observe tissue samples on the glass slides under a microscope.^8^ Such a traditional approach involves physical preparation and storage of glass slides and local consultations only, limiting the efficiency, reproducibility, scalability, and accessibility of the technique. These limitations, along with the advent of modern computers, imaging systems, and quantitative image processing techniques, have prompted a shift towards DP.^26^ The journey of digitization in pathology began with the development of the first virtual microscope during 1996-1998, where high-resolution images replicating the glass slides were produced using imaging scanners.^27^ The system was later advanced to support data caching and precomputed image pyramids.^27^ In the last two and half decades, the emergence and commercialization of WSI and glass slide scanners have revolutionized clinical pathology practices.^27^^,^^28^ Presently, many commercial WSI scanners are available (Philips, Leica, and Hamamatsu, etc), and many open-source WSI systems (caMicroscope, Digital Slide Archive, Sedeen viewer, QuPath) are supported actively.^29–32^ The advent of DP, powered by WSI, has revolutionized medical diagnostics.^26^^,^^33^ Improvements in technology by turning glass slides into digital formats and decreasing storage costs have increased the use of WSIs.^34^ The adoption of DP by pathologists has seen significant growth, increasing from 30% in 2013 to 64% in 2021, according to a report by the College of American Pathologists.^26^ In Figure 1, we have presented a template for the future roadmap for DP and CPATH that may have the necessary components and methods in a unified framework. With a multitude of advantages, DP has brought a revolution in high-resolution magnification analysis, electronic storage, automation of processes, quality assurance and control, telepathology and enhanced diagnostic efficiency, streamlined diagnostic workflow, accessible measurements and quantification, cancer prescreening, intraoperative consultation, histopathological second opinion, and education and training.

## Computational pathology—AI in DP

More recently, the advancements in AI and ML have started revolutionizing DP, which has been a helpful tool in histopathology image analysis in the last decade.^26^ Digital Pathology Association (DPA) defines CPATH as, “the big-data approach to pathology,” where patient’s pathology images and associated meta-data are combined to extract patterns and analyse image features through AI techniques.^6^ To address the evolving landscape of pathology practice and the increasing adoption of digital tools in diagnostic workflows, the College of American Pathologists in consultation with the American Medical Association, announced the inclusion of 30 new current procedural terminologies (CPTs) related to DP for use by pathologists, effective from January 1, 2024.^35^ The integration of AI in DP has facilitated precise diagnosis by processing information that may not be perceived by the human eye at times.^8^^,^^27^ The cardinal tasks of AI in DP include the development and validation of models and algorithms that can detect different patterns and features from data presented in the form of WSIs. The AI models can identify regions of interest (ROIs) from WSIs, extract and classify patches, classify entire WSIs, generate labels, and identify various objects of interest.^27^ Examples of how various AI and ML models have been proposed to advance various DP tasks include, diagnosis support, enhanced image analysis, improving prognostics and biomarkers predictions, post-treatment analysis and disease surveillance, and cross-modality learning.^6^^,^^8^^,^^19^^,^^26^^,^^36^ The key terminologies used in AI are presented in Box 2.

**Box 2.** Key AI terminologies.

AI	Initially started as a simple theory of human intelligence being exhibited by machines, AI aims to simulate human intelligence in machines by developing computers that can engage in learning, reasoning, self-correction, problem-solving, and decision-making.^37^
ML	ML is a subset of AI that programs computers to process natural data and solve an optimization problem using data samples or past experience. It is an engineering discipline that is closely related to mathematical statistics to build learning models.^38^
Deep Learning (DL)	DL is a subfield of ML and refers to models developed using multiple layers of artificial neural networks. The deep neural networks focus on compositional learning using multiple (hence the word “deep”) layers of representations, where each layer is built using multiple artificial neurons.^38–42^ Various types of DL models have been developed, including Convolutional Neural networks (CNNs), Recurrent Neural networks (RNNs), Graph Neural Networks (GNNs), Transformers, and many others.
Convolutional Neural Networks	CNNs are specialized DL models for analysing image or grid data.^38^^,^^43^^,^^44^ CNNs use convolution operations and can learn spatial correlations, features, and relationships from the input data.^43^^,^^45^
Recurrent Neural Networks	RNNs are a family of DL models used for processing sequential data like speech, text, or time-series data (eg, patient vital signs recorded over time).^43^^,^^46^^,^^47^ RNNs are used to model time/space-based relationships between sequences of input data and to learn patterns based on the order (in time or space).^43^ Sub-categories of RNNs called Long short-term memory network (LSTM) and gated recurrent unit (GRU) models are better at learning long-term dependencies, making them great for real-world use cases, eg, predicting medical events and detecting anomalies in time series data.^46^
Graph Neural Networks	GNNs are DL models designed for analysing graph data, like social relationships or biological pathways.^40^ GNNs pass messages between neighbouring nodes to aggregate local and global information. By modelling these relationships, GNNs can make predictions about nodes, edges, and sub-graphs in the network, where each entity may represent patients, drugs, proteins, etc GNNs enable new insights in fields like precision medicine and drug discovery.^48–50^
Transformers	Transformers are DL models that have shown promising results on language translation and image recognition tasks.^51^^,^^52^ Their key innovation is a technique called self-attention. Unlike RNNs, which process data sequentially, self-attention allows Transformers to learn relationships between any parts of the input, regardless of their position in the sequence. For example, a Transformer can learn that the symptoms “cough” and “fever” are strongly related even if they are distant in the patient’s medical history. The model learns which connections are important by assigning an “attention” score between input sequences (also referred to as tokens).^47^^,^^51^^,^^52^
Foundation Models (FMs)	FMs are a new class of DL models characterized by their large scale and ability to adapt to new tasks.^53^ FMs are trained on extensive datasets, allowing them to develop a broad understanding of various topics.^8^^,^^54^ The key characteristics of FMs include scalability in learning from diverse data sources, multimodal learning, compositionally in terms of generalization, and the emergence of implicit learning. Transfer learning, adaptation, in-context learning, and fine-tuning make FMs exceptionally useful across numerous fields, including the medical domain.^55^ A recent review paper covers existing FMs and their prospective use in DP.^8^
Multimodal Learning	Multimodal Learning involves integrating data from multiple modalities for an ML task, eg, prediction, classification, or regression. A modality refers to specific data input or source types, such as text, images, audio, video, or other data. The core idea is to leverage the complementary information from different modalities to improve the performance and accuracy of learning algorithms. Multimodal learning aims to achieve a more comprehensive understanding of the problem, leading to better decision-making than using a single modality.^19^^,^^56^

## DP, CPATH, and multimodal AI

Advancements such as the digitization of patient health records, the development of new diagnostic technologies, innovative laboratory tests, and the establishment of robust information technology infrastructure in hospitals have led to an exponential increase in the volume of medical data collected for each patient.^19^ Recent progress in multimodal learning, driven by deep neural networks, has demonstrated a remarkable ability to learn from varied data modalities, such as computer vision and natural language processing.^19^^,^^53^ Multimodal foundation models such as OpenAI’s Contrastive Language-Image Pretraining (CLIP) and Generative Pretraining Transformer (GPT-4) have established new standards in the field, while the Foundational Language And Vision Alignment Model (FLAVA) marks a notable advancement by integrating vision and language representation learning to enable multimodal reasoning.^57–59^ The success of multimodal AI models in other domains has inspired cancer researchers to explore the potential of multimodal AI in healthcare.^60^ The multimodal AI models being developed in the medical domain can integrate diverse modalities, including radiological images, genetic profiles, and clinical information, within the DP workflow, enhancing diagnostic precision and enabling personalized treatments.^61^^,^^62^ Multimodal AI models are poised to empower healthcare teams by offering them a holistic view of the patient’s disease state and paving the way for truly personalized cancer care.^9^^,^^54^^,^^61^

In our context, “modality” refers to a specific data source or type. For example, EHR text descriptions of surgical procedures or post-visit reports represent one modality. Lab test values, vital measurements, and demographic information form another. Histopathology slides (stored as WSI), radiological scans (X-ray, MRI, PET, CT), and molecular data (genomics, proteomics) are separate modalities.^39^^,^^62^^,^^63^ Some modalities have sub-categories, such as MRI scans with FLAIR, T1, T2, and CT scans with various views and contrast protocols.

In multimodal learning, researchers use various modality-specific data processing methods and data fusion mechanisms. The generally defined 5 main stages are: (1) preprocessing, (2) feature extraction, (3) data fusion, (4) primary learner, and (5) final classifier.^19^ Data fusion from different modalities can occur at various levels. Early fusion combines features before feeding data into ML models.^62^^,^^64^ Intermediate fusion occurs at the model level, while late fusion merges processed data at the decision level.^19^ Explainability in deep learning involves techniques to make models’ decisions, behaviours, and outputs transparent and understandable.^65^^,^^66^ This includes building interpretable models or generating *post hoc* explanations.^8^^,^^65^ In DP, which uses high-resolution WSIs, explainability is crucial. Techniques include heatmaps showing areas of significance, case-based explanations comparing with established predictions, and layer-wise relevance propagation to trace outputs back to inputs.^67^

**Figure 2.**
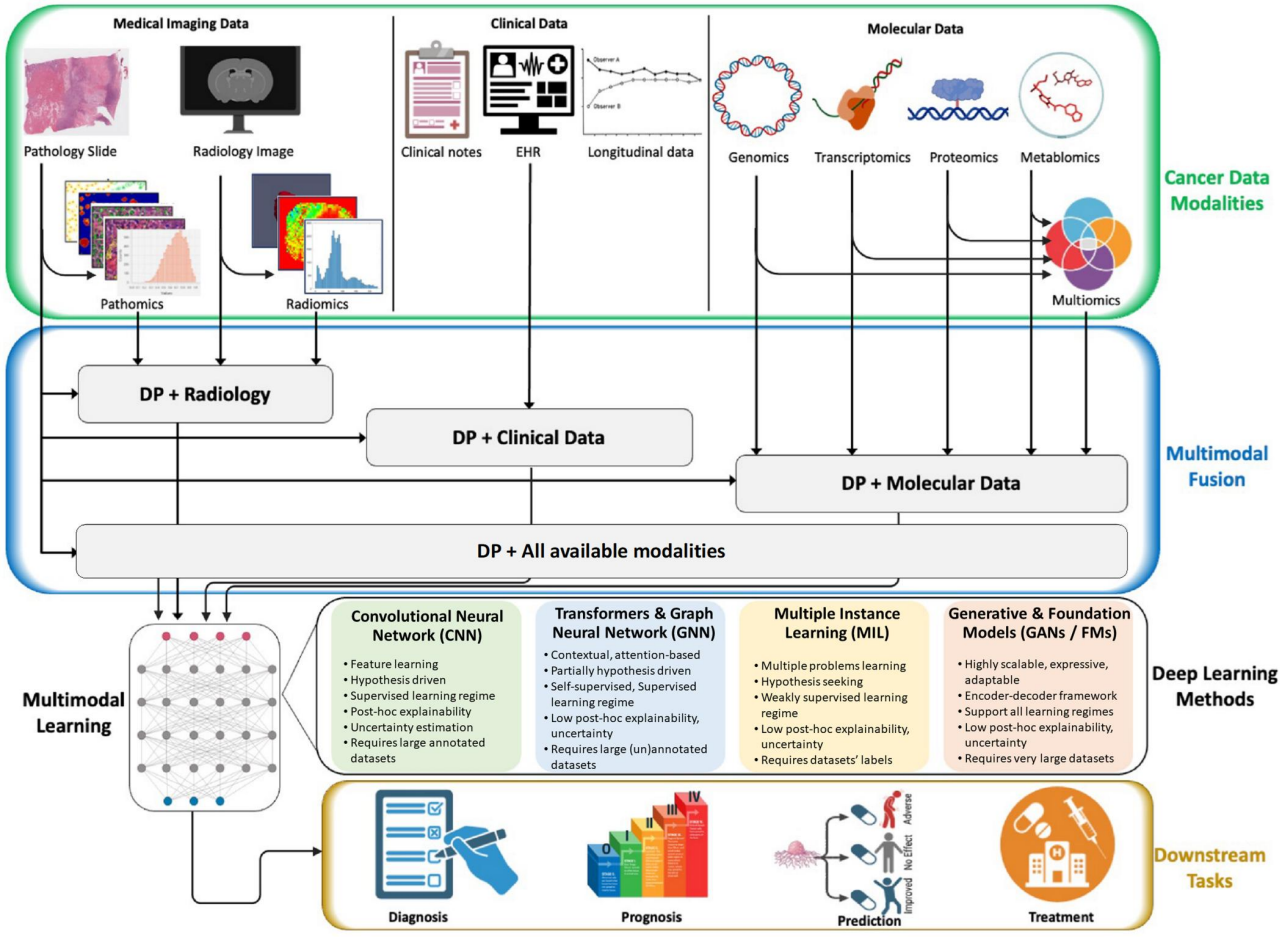
A schematic layout of multimodal learning in cancer care is presented. Multiple data modalities include histopathology images and data (including features extracted from gigapixel images referred to as pathomics features), radiological images and radiomics features (extracted from radiology images), clinical information, and molecular data. Various data modalities can be fused together by AI models to perform a range of downstream tasks, including disease diagnosis, prognosis, prediction, treatment planning, and post-treatment surveillance and monitoring. Data fusion can be performed at different levels and in many different ways using AI models only or a mixture of manual processing and AI models.

In the following, we provide a chronological overview of various applications of multimodal AI/ML techniques with a focus on histopathology data as the essential ingredient integrated with one or more other modalities (eg, radiology, genomics, or clinical data). A schematic layout of the multimodal AI in DP and CPATH is provided in Figure 2. We also provide a summary of references for each category of multimodal learning in Table 1, along with the explainability method of these works where applicable.

**Table 1.** Summary of multimodal AI models for DP reported in this review.

Modalities	Ref	Data type	Metrics	# patients	Task	Cancer type	Explainability
Pathology, Molecular	^68^	H&E, Multiomics (mutation status, CNV, RNAseq expr)	5-fold cv, *C*-index, AUC	5720	Survival outcome prediction and prognostic features correlations.	14 types (BLCA, BRCA, COADREAD, HNSC, KIRC, KIRP, LGG, LIHC, LUAD, LUSC, PAAD, SKCM, STAD, UCEC)	heatmaps, integrated gradients
	^69^	H&E, Multiomics (mutation status, CNV, RNAseq expr)	15-fold cv, *C*-index	Glioma: 769, CCRC: 417	Survival outcome prediction.	Glioma (TCGA-GBM, LGG), CCRCC (TCGA-KIRC)	heatmaps
	^18^	WSIs, genomic features	5-fold cv, *C*-index	3523	OS	5 types (BLCA, BRCA, GBMLGG, LUAD, UCEC)	heatmaps
	^70^	WSIs, Multiomics (DNA mutation, mRNA expr)	AUC	1214	Microsatellite status prediction	TCGA-COAD, Asian-CRC	correlation heatmaps
	^71^	H&E slides, spatially resolved gene expression	Accuracy	7	Cancer detection, predict spatial variation of individual genes	Prostate cancer	heatmaps
	^72^	WSI, Multiomics (genomics, proteomics, and molecular subtyping)	*C*-index	1888	Survival prediction, MSI prediction	Colorectal cancer (TCGA-COAD, TCGA-READ)	importance scores, heatmaps
Pathology, Clinical	^73^	WSIs, Pathomic features, Clinical	10-fold cv, Accuracy, AUC	735	Tumour grade prediction	TCGA-LGG, TCGA-HGG	no
	^74^	Dermatology images, Clinical	Balanced accuracy, AUC	>3300	Cancer classification	Skin cancer	no
Pathology, Radiology	^75^	WSI, MRI	NPV, PPV, accuracy, AUC, Cohen’s kappa	400	Tumour classification 1) benign/cancerous, 2) high-risk/low-risk tumour	Prostate cancer	class activation heatmaps
	^76^	Radiomics (from MRI), Pathomic features (from H&E)	Spearman’s correlation	48	Association among radiomics and pathomics features	Glioblastoma multiforme (CPTAC-GBM)	no
	^87^	MRI (T1-GD, T1, T2, and T2-FLAIR), H&E images	5-fold cv, Regression	171	Survival prediction	Gliomas (TCGA-HGG, LGG)	no
	^88^	MRI (T2, T1-CE, DWI), H&E images	AUC, PPV, NPV	Retrospective study: 933,Prospective study: 100	Predict pathological complete response	Rectal cancer	no
	^89^	MRI (T2WI, DWI), H&E images	AUC	153	Predict pathological good responses	Rectal cancer	no
	^77^	CT, H&E images	AUC, accuracy	171	Cancer classification	NSCLC	heatmaps
	^78^	CT, WSIs	AUC, accuracy	252	Prediction of LNM stage	Gastric cancer	class activation maps
Pathology, Radiology, Molecular	^79^	Radiology (CT), pathology (IHC), Multiomics (somatic mutations, CNAs, gene fusions)	10-fold cv, AUC	247	Immunotherapy response (IHC score, TMB)	NSCLC	no
Pathology, Radiology, Clinical	^80^	Pathomics, Radiomics features, Immunoscore, Clinical factors	AUC	103	Survival prediction	Colorectal cancer (CRC) lung Metastasis	no
	^81^	Pathomics, Radiomics, Clinicopathological features	AUC	211	Predicting pathological complete response	Breast cancer	no
Pathology, Molecular, Clinical	^82^	WSIs, Multiomics (mRNA, miRNA expr), Clinical	*C*-index	11 160	OS	20 types (BLCA, BRCA, CESC, COADREAD, HNSC, KICH, KIRC, KIRP, LAML, LGG, LIHC, LUAD, LUSC, OV, PAAD, PRAD, SKCM, STAD, THCA, UCEC)	no
	^83^	WSIs, Multiomics (CNA, somatic genetic mutations), Clinical	AUC	1281	Detect malignancy, Diagnose cancer subtypes, Predict survival outcomes	CCRCC	grad-CAM
	^84^	H&E, Multiomics (genomic and transcriptomic features), Clinical	AUC	168	Response to treatment	Breast cancer	no
Pathology, Radiology, Molecular, Clinical	^90^	H&E, CT, Multiomics (genomic features), Clinical	*C*-index	444	OS	Ovarian cancer	correlation heatmap
	^85^	H&E, MRI, Multiomics (DNA seq), Clinical	15-fold Monte Carlo cv, *C*-index	176	OS	Glioma (TCGA-GBM, LGG)	no

Refer to Table 2 for the list of abbreviations used here.

**Table 2.** List of abbreviations used in this article, alphabetically ordered.

AI	Artificial Intelligence	AP	Anatomical Pathology
AUC	Area Under the Curve	BLCA	Bladder Urothelial Carcinoma
BRCA	Breast Invasive Carcinoma	*C*-Index	Concordance Index
CCRCC	Clear Cell Renal Cell Carcinoma	CI	Confidence Interval
CNAs	Copy Number Alterations	CNNs	Convolutional Neural Networks
CNV	Copy Number Variation	COAD	Colon Adenocarcinoma
COADREAD	Colorectal Adenocarcinoma	CP	Clinical Pathology
CPATH	Computational Pathology	CPT	Current Procedural Terminologies
CPTAC	Clinical Proteomic Tumour Analysis Consortium	CRC	Colorectal Cancer
CT	Computed Tomography	CV	Cross Validation
DFS	Disease Free Survival	DL	Deep Learning
DLRPM	Deep Learning RadioPathomics Model	DNA	Deoxyribonucleic Acid
DOF	Deep Orthogonal Fusion	DP	Digital Pathology
DPA	Digital Pathology Association	EHR	Electronic Health Records
EPLA	Ensembled Patch Likelihood Aggregation	FFPE	Formalin-Fixed Paraffin-Embedded
FHIR	Fast Healthcare Interoperability Resources	FLAIR	Fluid Attenuated Inversion Recovery
FMs	Foundation Models	FNA	Fine Needle Aspiration biopsy
GANs	Generative Adversarial Networks	GBM	Glioblastoma Multiforme
GBMLGG	Glioblastoma & Lower Grade Glioma	GNNs	Graph Neural Networks
GRU	Gated Recurrent Unit	H&E	Haematoxylin and Eosin
HER	Human Epidermal Growth Factor Receptor	HGG	High Grade Gliomas
ID	Integrative Diagnostics	ICB	Immune Checkpoint Blockade
IHC	Immunohistochemistry	JIF-MMFA	Joint-Individual Fusion with Multi-modal Mutual Fusion Attention
KIRC	Kidney Renal Clear Cell Carcinoma	LGG	Low Grade Gliomas
LNM	Lymph Node Metastasis	LSTMs	Long Short-Term Memory Network
LUAD	Lung Adenocarcinoma	LUSC	Lung Squamous Cell Carcinoma
MCAT	Multimodal Co-Attention Transformer	MIL	Multiple Instance Learning
miRNA	Micro-Ribonucleic Acid	ML	Machine Learning
MMF	Multimodal Fusion	MMO	Multimodal Orthogonalization
MOMA	Multi-Omics Multi-cohort Assessment	MRI	Magnetic Resonance Imaging
mRNA	Messenger-Ribonucleic Acid	MSI	Microsatellite Instability
nCRT	Neoadjuvant Chemoradiotherapy	NGS	Next-Generation Sequencing
NPV	Negative Predictive Value	NSCLC	Non-Small Cell Lung Carcinoma
OS	Overall Survival	OV	Ovarian Carcinoma
PAAD	Pancreatic Adenocarcinoma	PCR	Polymerase Chain Reaction
PD-L1	Programmed Death-Ligand 1	PET	Positron Emission Tomography
pGR	Pathological Good Response	PI-RADS	Prostate Imaging Reporting And Data System
PPV	Positive Predictive Value	QA	Quality Assurance
QC	Quality Control	RAPIDS	RAdioPathomics Integrated preDiction System
ResNet	Residual Network	RNA-Seq	Ribonucleic Acid Sequence
RNNs	Recurrent Neural Networks	SNNs	Self-Normalizing Networks
SVM	Support Vector Machine	TCGA	The Cancer Genome Atlas
TMB	Tumour Mutation Burden	TME	Tumour Microenvironment
UCEC	Uterine Corpus Endometrial Carcinoma	ViT	Vision Transformer
WHO	World Health Organization	WSI	Whole Slide Imaging

### 2019

Cheerla et al. developed a multimodal model to predict the survival of patients for 20 different cancer types by fusing three different data modalities that included clinical data, mRNA and microRNA expressions, and WSIs.^82^ The proposed multimodal AI model was able to predict the overall survival (OS) with the concordance index (*C*-index) of 0.78.^82^

### 2020

Chen et al. proposed an end-to-end multimodal framework called “Pathomic Fusion” that predicted survival outcomes from WSIs and genomic (mutations, CNV, RNA-Seq) features of glioma and clear cell renal cell carcinoma (CCRCC).^69^ For the glioma patients, Pathomic Fusion outperformed the WHO paradigm and previous state-of-the-art with 6.31% and 5.76% improvements, respectively, and reaching a *C*-index of 0.826.^69^ For the CCRCC, similar results were observed, with tri-modal Pathomic Fusion achieving a *C*-index of 0.720.^69^

Microsatellite Instability (MSI) is an approved pan-cancer biomarker for immune checkpoint blockade (ICB) therapy. A multiple-instance AI model, called Ensembled Patch Likelihood Aggregation (EPLA), was proposed to integrate pathological, genomic, and transcriptomic phenotypes and predict MSI.^70^ The model also provided an interpretation of its results for effective use in ICB therapy. The proposed EPLA model got an AUC of 0.8848 and 0.8504 in The Cancer Genome Atlas (TCGA)-COAD and Asian-CRC external validation, respectively.^70^ EPLA was also able to capture correlation between poor differentiation and MSI.^70^ The model was also able to identify imaging signatures associated with mutations burden, DNA damage, and antitumour immunity.^70^

Rathore et al. used support vector machine (SVM) model to distinguish high-grade gliomas (HGG) from low-grade gliomas (LGG) by combining textual features with conventional imaging and clinical markers.^73^ Their model identified the microvascular proliferation level, mitotic activity, presence of necrosis, and nuclear atypia in WSIs.^73^ The texture features were successfully validated on the glioma patients in 10-fold cross-validation (accuracy = 75.12%, AUC = 0.652). Adding texture features to clinical and conventional imaging features significantly improved grade prediction compared to the models trained on clinical and conventional imaging features alone.^73^

Classical ML models have been shown to capture the multi-scale association between H&E and CT images, which was used to classify lung adenocarcinoma (LUAD) from squamous cell carcinoma in NSCLC.^77^ Alvarez et al. identified discriminant pathomic patterns for cancer classification and established correlations between multimodal features of 171 patients, depicting significant cross-scale associations between cell density statistics and CT intensity.^77^

### 2021

The Multimodal Co-Attention Transformer (MCAT), an AI model leveraging the Transformer architecture and cross-modality attention, has outperformed all unimodal models and the more commonly used late fusion methods in survival prediction for 5 distinct types of cancers, including Bladder Urothelial Carcinoma (BLCA), Breast Invasive Carcinoma (BRCA), Glioblastoma & Lower Grade Glioma (GBMLGG), LUAD, and Uterine Corpus Endometrial Carcinoma (UCEC).^18^ MCAT outperformed state-of-the-art methods on all benchmarks, with a *C*-index increase of 6.35% and 12.4% compared to Attention Multi-Instance Learning (MIL) and Deep Attention Multiple Instance Survival Learning (DeepAttn-MISL) methods, respectively. The success of the MCAT model indicated that multimodal learning could be used in general for any survival outcome prediction task.^18^

Khosravi et al. proposed AI-biopsy, an AI model based on the Inception-V1 architecture, which was pretrained on a non-medical ImageNet dataset and transfer learned on MRI scans labelled with pathology assessments like Gleason scores and grade groups.^75^ The proposed early data-fusion technique exhibited a higher agreement with biopsy results when compared to the Prostate Imaging Reporting and Data System (PI-RADS) score, indicating promising advancements in diagnostic precision.^75^ AI-biopsy achieved AUCs of 0.78 (95% CI: [0.74-0.82]) and 0.89 (95% CI: [0.86-0.92]) in 400 patients on the tasks of distinguishing high-risk from low-risk cancers and cancerous vs benign prostate disease, respectively.

WSI, demographic, genomic, and clinical data collected from 3 data sources, TCGA, Clinical Proteomic Tumour Analysis Consortium (CPTAC), and Brigham and Women’s Hospital, were used to develop an AI model for various tasks related to renal cell carcinoma.^83^ The trained models were able to detect malignancy (AUC = 0.964-0.985), diagnose renal cancer subtypes (AUCs = 0.953-0.993), and predict survival outcomes.^83^ The model also identified histopathology image features that were indicative of biomarkers such as copy-number alterations (CNAs) and tumour mutation burden (TMB).^83^

In general, single-omics data modality may not be able to provide a comprehensive view of tumour-related molecular alterations. In such cases, integrating multiple data modalities even within the -omics regime can help improve diagnosis, personalized treatments, and predict outcomes more accurately.^86^ Tong et al. developed an encoder-decoder AI model for cancer classification among 4 cancer types [LUAD, Kidney Renal Clear Cell Carcinoma (KIRC), Lung Squamous Cell Carcinoma (LUSC), and pancreatic adenocarcinoma (PAAD)] and patient survival prediction in 2 cancer types (BRCA, Ovarian Carcinoma [OV]).^86^ Two data integration methods were evaluated for 4 types of -omics data that include gene expression, DNA methylation, miRNA expression, and copy number variations.^86^ The multimodal AI models outperformed single-omics models for both tasks, cancer classification and survival prediction. Within the 2 data fusion methods, divergence-based consensus networks performed better than concatenation-based integration networks.^86^

Multimodal learning using clinical data, H&E images, genomics, and transcriptomic features were shown to predict pathological complete response to treatment and improve outcomes in 168 breast cancer patients treated with chemotherapy with or without HER2-targeted therapy before surgery.^84^ The multiomic AI model predicted pathological complete response in an external validation cohort of 75 patients with an AUC of 0.87. The final AI model consisted of an ensemble of traditional ML models, including logistic regression, SVM, and random forest.^84^

Chelebian et al. developed an ensemble CNN to provide interpretations that matched pathologists’ manual assessments.^71^ The resulting interpretations were more informative when the underlying genes were considered.^71^ The framework paired H&E slides with spatially resolved gene expressions from the spatial transcriptomics technique, predicting the spatial variation of individual genes.^71^

Braman et al.^85^ proposed a Deep Orthogonal Fusion (DOF) model to learn from multiparametric MRI scans, H&E slides, DNA sequencing, and clinical features into a multimodal risk score.^85^ The embeddings (or representations) from individual data modalities were learned and fused through attention-gated tensor fusion. The authors introduced multimodal orthogonalization (MMO) learning loss that incentivized each embedding to be more complementary.^85^ DOF predicted OS in 176 glioma patients with a median *C*-index of 0.788 ± 0.067, compared to the best-performing unimodal model (*C*-index of 0.718 ± 0.064).^85^

### 2022

Compared with unimodal data, combining H&E WSIs with the molecular assay improved survival prediction reliability in multiple cancers, including BRCA, COADREAD, LUAD, PAAD, and others.^68^ Chen et al. proposed a deep learning framework, multimodal fusion (MMF), which achieved an overall *C*-index of 0.644 across the 14 cancer types, whereas the MIL model using WSI and SNN on molecular data had overall *C*-Indices of 0.578 and 0.606, respectively. On survival AUC, a similar improvement for the multimodal model was reported with an overall performance of 0.662 compared with unimodal models.^68^

Often referred to as a “virtual biopsy,” radiomics features enhance conventional diagnostic radiologic workflows when combined with the pathomic features from H&E slides.^76^ Brancato et al. employed different feature generation techniques to extract 91 radiomics features from MRI scans and 156 pathomic features from H&E images for 48 Glioblastoma multiforme (GBM) patients from the CPTAC data portal. The proposed radiomics-pathomics fusion technique showed significant cross-scale associations of features among the GBM patients.^76^

Multimodal integration of radiology, pathology, and genomics data modalities improved insights into response to immunotherapy PD-L1 blockade in patients with NSCLC.^79^ Vanguri et al. explored the multimodal integration of CT images, digitized programmed death ligand-1 IHC slides, genomics data including somatic mutations, copy number alterations and gene fusions, and known outcomes to immunotherapy to predict immunotherapy response among 247 patients with advanced NSCLC. The multimodal model called DyAM showed an AUC of 0.80, 95% CI [0.74-0.86] on immunotherapy response prediction task, that outperformed unimodal methods, including TMB (AUC = 0.61, 95% CI [0.52-0.70]) and programmed death ligand-1 IHC score (AUC = 0.73, 95% CI [0.65-0.81]).^79^

Ensemble of a range of ML regression models, including support vector regression, AdaBoost, gradient boost, and random forest, were shown to provide better survival outcomes prediction for 171 Glioma patients using integrated features from MRI (T1-GD, T1, T2, and T2-FLAIR) and H&E images.^87^ The proposed ensemble fusion approach outperformed several other techniques based solely on radiology features, gene expression data, or pathology images.^87^

Combining pretreatment MRI (T2, T1-CE, DWI) and H&E-stained biopsy slides using AI models can predict pathological complete response in patients with locally advanced rectal cancer.^88^ The proposed framework, RAdioPathomics Integrated preDiction System (RAPIDS), showed a significant improvement in the prediction of pathological complete response with a combined AUC of 0.812 compared to the unimodal models for prospective validation study.^88^ RAPIDS also performed better in the retrospective study training cohort (AUC 0.868 [95% CI 0.825-0.912]), validation cohort 1 (0.860 CI [0.828-0.892]), and validation cohort 2 (0.872 CI [0.810-0.934]).^88^

Harnessing AI-driven insights with radiomics–pathomics has led to personalized treatment plans, optimizing therapies while minimizing adverse effects.^80^ An innovative fusion of pathomics, radiomics, and immunoscore data identified high-risk patients with colorectal cancer (CRC) lung metastasis. The combined nomogram model predicted the OS with an AUC of 0.860 and disease-free survival (DFS) with an AUC of 0.875. This integration offers insights about aggressive treatment strategies and tailored follow-up plans for high-risk patients.^80^

Wan et al. developed a multiscale framework that harnessed the power of radiomics features extracted from MRI (T2WI, DWI) data, pathomics features derived from H&E images, and clinical variables to predict pathological good responses (pGR) in 153 patients with locally advanced rectal cancer who underwent neoadjuvant chemoradiotherapy (nCRT).^89^ This macroscopic–microscopic scale fusion identified the patients with down-staging to stage ypT0-1N0 after nCRT, a critical factor in guiding organ-preserving strategies to minimize invasion and uphold the quality of life. By integrating these diverse modalities, the multiscale AI model outperformed the traditional clinical-radiological model with an AUC of 0.90 (95% CI: [0.78-1.00]) compared to 0.68 (95% CI: [0.46-0.91]) in the validation group. These results highlight the complementary nature of information present in radiomics and pathomics features for assessing treatment response.^89^

Boehm et al. used a multimodal dataset of 444 patients with ovarian cancer and integrated radiological data (CT scans), histopathological images (H&E), clinical, and genomic modalities to stratify patients based on risk.^90^ The multimodal AI model stratified patients in the training and the test cohorts with the *C*-indices of 0.55 (95% CI: [0.549-0.554]) and 0.53 (95% CI: [0.517-0.547]), respectively. The authors also discovered quantitative features, such as tumour nuclear size on H&E and omental texture on CT, associated with prognosis.^90^

### 2023

Palmal et al. employed a graph convolutional network (GCN) and Choquet fuzzy ensemble consisting of classical ML models (Logistic Regression, Random Forest, and SVM) to fuse the multiomics and clinical data.^91^ 1980 samples of breast cancer patients were classified as short-term or long-term survivors using different combinations of gene expression (24 000 features), copy number alteration (26 000 features), and clinical data (27 features).^91^ The performance metrics of accuracy, Matthews correlation coefficient, precision, sensitivity, specificity, balanced accuracy, and F1-measure showed significant result scores as 0.820, 0.528, 0.630, 0.666, 0.871, 0.769, and 0.647, respectively.^91^

Tsai et al. developed a Multi-omics Multi-cohort Assessment (MOMA) framework to fuse WSIs with genomics, proteomics, and molecular data to predict OS, progression-free survival, and other variables for colorectal cancer patients.^72^ MOMA was able to identify cross-scale relationships among histologic patterns, multiomics, and clinical profiles in 1888 patients. MOMA predicted OS, progression-free survival, and MSI in held-out test datasets of stage I, II, and III colorectal cancer patients better than other state-of-the-art methods.^72^

An AI model that fuses CT images with WSIs was shown to accurately predict lymph node metastasis stage (LNMs) in patients with gastric cancer.^78^ Features extracted from CT images using a CNN (ResNet-50) and from WSI using the Vision Transformer model (ViT) were fused to learn the prediction task of 5 LNM stages.^78^ The proposed radiopathologic integrated method achieved 84.0% accuracy and significantly improved AUCs in predicting five LNM stages compared to single-modality models.^78^ The integrated model also achieved an AUC of 0.978 (95% CI: [0.912-1.0]) in metastasis prediction task.^78^

An AI model called Deep Learning Radio-Pathomics Model (DLRPM) seamlessly integrated radiomics, pathomics, clinical, and pathological features using ResNet-50 and SVM on 211 breast cancer patients who underwent neoadjuvant chemotherapy.^81^ DLRPM demonstrated remarkable capabilities for predicting pathological complete response in the validation dataset with an AUC of 0.927 (95% CI: [0.858-0.996]), significantly outperforming models that used individual data modalities and features, such as radiomics and pathomics.^81^

The fusion of radiomics features with pathomics features and molecular data may provide a more comprehensive view of the intricate tumour microenvironment (TME), enabling a deeper understanding of its composition and tumour heterogeneity.^92^ Fusing radiological and pathomics data may offer precise spatial mapping of histological features, facilitating detailed TME examination. This holistic approach may unravel the complex interactions between tumour cells and the surrounding microenvironment, advancing our understanding of cancer biology.^92^

Recently, Tang et al. designed a new fusion method to combine dermatological images and patient clinical metadata for skin cancer classification using three public skin lesion datasets with more than 3300 patients.^74^ The proposed AI model, called joint-individual fusion with multimodal mutual fusion attention (JIF-MMFA), learns the shared features of multimodal data by employing a fusion attention module to enhance the relevant features from both data modalities.^74^ JIF-MMFA showed the effectiveness of using patients’ clinical data in improving the classification of skin cancer.^74^

## Discussion

The global market for AI-based DP solutions is projected to grow at a compound annual growth rate of 8.59% from 2024 to 2031, with North America leading due to advanced healthcare infrastructure, higher income levels, and research facilities.^93^ AI patent filings in DP have sharply increased in major patent offices, indicating growing innovation.^94^ There is a significant push towards open-source and reproducible AI, with initiatives like Allen AI contributing through platforms like Hugging Face.^95^ AI is increasingly used to predict patient responses to immunotherapy by analysing histopathological and molecular data.^96^ The integration of AI in DP promotes collaboration among pathologists, oncologists, radiologists, and bioinformaticians, leading to a holistic approach to patient care.^97^

Large language models (LLMs) like ChatGPT enhance pathology reports by ensuring consistency and translating complex medical data for clinicians and patients. LLMs assist pathologists by providing quick access to relevant literature and summarizing research findings.^8^^,^^98^ While most multimodal works focus on 2D WSIs, incorporating 3D data from techniques like microscopy or medical imaging could provide additional spatial context, improving model performance.^99^ Combining WSIs with text reports, genomic data, or medical videos in multimodal learning is emerging as a way to enhance diagnostic capabilities.^100^ Models like CLIP, CONCH, which integrate text and image data, can be adapted for DP tasks.^55^ Integrating histopathological, radiomic, genomic, and clinical data using a late-fusion approach has been shown to improve cancer patient risk stratification.^90^ Multimodal AI is used across various domains, including biomedicine for disease diagnosis and treatment planning, autonomous vehicles for real-time decision-making, and augmented generative AI for content creation and multimodal translation.^101–105^ Below we discuss some of the opportunities and challenges of multimodal learning in DP.

## Opportunities and challenges

### Opportunities

*Multimodal patient stratification*: Integrating various data types, including genomics, transcriptomics, epigenomics, proteomics, histopathology, and radiology, allows more precise patient stratification.^17^ AI models can identify distinct patient subgroups with different phenotypes and treatment responses. This stratification can lead to more personalized and effective treatment strategies, enhancing patient care.

*Non-invasive alternatives, personalized medicine*: AI models enable the prediction of histology subtypes or grades from radiomics features.^106^ This capability will open many opportunities for developing non-invasive surrogates for existing biomarkers, reducing the need for invasive procedures in the future. Additionally, AI models demonstrate promise in predicting clinical outcomes, including survival, treatment response, and recurrence, enabling personalized medicine approaches and better patient management.

*Patient empowerment*: Integrating different modalities can create user-friendly digital solutions that empower patients to become active partners in managing their health.^56^ Patients can access their own health records, diagnostic reports, and integrated biomarker information, allowing them to make informed decisions about their care. This level of patient engagement and access to integrated health information can enhance the patient experience, improve communication with healthcare providers, and improve health outcomes.

*Improving outcomes*: Multimodal data integration, especially fusing histopathology and genomics data, has shown promise in improving the accuracy of outcome predictions for cancer patients.^17^ Researchers and clinicians can improve overall/progression-free survival analysis by leveraging multimodal AI techniques, enabling more precise prognostication beyond traditional clinical factors. This can facilitate the development of more effective treatment regimens for high-risk patients and improve treatment outcomes.

*Morphological associations, biomarker discovery*: Multimodal AI models can potentially discover morphological associations spread across data modalities, including the relationship between genetic mutations and tissue morphology.^106^ Such findings can help exploratory studies and reduce the search space for potential biomarker candidates. By identifying morphological associates, AI can support cost-efficient biomarker discovery and aid in cancer screening and therapeutic target discovery.

*Integrative diagnostics*: Integrative diagnostics (ID) is defined to be the convergence of radiology and pathology imaging, clinical laboratory medicine, and advanced computational methods.^107^ ID, especially with a focus on radiology and pathology data fusion, offers many benefits and untapped potential in oncology. Yet, despite the anticipation of numerous benefits, a noticeable scarcity of comprehensive literature surrounds this emerging field. ID can potentially identify appropriate therapies and suggest treatment changes when needed, decreasing morbidity and costs and improving patient outcomes.^107^

*Correlational studies*: Multimodal learning may hold enormous potential for conducting correlational studies and improving oncology patient diagnoses. Using histopathological and molecular data, AI models have already been shown to identify novel biomarkers and underlying tuberculosis mechanisms in susceptible individuals.^108^

*Medical report integration*: Despite recent advancements related to digitizing medical data in EHR and radiological and laboratory imaging systems, there are apparently no efforts focused on correlating the imaging data (histopathology and radiology) with the textual reports (eg, radiology reports, pathology reports, and other clinical notes) at the data storage level. One obvious reason is related to the siloed storage and data management systems. Modern AI systems based on vector databases can provide the necessary building blocks for data integration at the storage level.^109^

### Challenges

*Data availability, integration, missingness*: Obtaining and integrating diverse medical data types, such as images and clinical records, is an extremely challenging task.^110^ Data security and governance issues, data scarcity, unstructured formats, and varying data identifiers pose significant hurdles in this process.^17^^,^^92^ Moreover, the issue of missing or incomplete data within each data modality further complicates the training of AI models.^106^^,^^110^ Strategies like synthetic data generation, dropout-based methods, and embedding models are being explored to address this challenge, but concerns about the accuracy and representativeness of synthetic data still remain.^62^^,^^64^

*Interoperability*: Ensuring that different medical devices, systems, and data formats can seamlessly communicate and exchange information is a considerable challenge.^56^^,^^91^ Data standards, protocols, and technologies differ across vendors, providers, and sometimes healthcare institutions and systems. Efforts such as Fast Healthcare Interoperability Resources (FHIR) for the EHR to overcome this challenge are essential for integrating diverse modalities and facilitating comprehensive patient care.

*Data modelling and analysis*: This challenge arises when integrating data from different sources or institutions because of institution-specific biases stemming from variations in data acquisition methods, clinical data standards, and the catchment area demographics.^17^ Ensuring model generalizability in the presence of heterogeneous data modalities is another aspect of this challenge.^17^

*Transparency and clinical adoption*: While multimodal AI models hold great promise in oncology, their adoption in clinical practice faces several hurdles. Ensuring transparency and interpretability of these models is challenging, as they often use abstract feature representations.^106^ Prospective clinical trials and rigorous validation studies will be needed to demonstrate the added value of AI models in real-world clinical settings. Regulatory guidelines and addressing issues like fairness and dataset shifts are equally important for clinical adoption.^106^

*Personnel training*: The primary users of AI systems in DP are clinicians and pathologists. Training the administrative, technical, and professional personnel on AI systems is a secondary but essential challenge that needs to be addressed for the success of AI systems.^111^

*Explainability, trust, reproducibility, and output visualization of AI models*: AI models are known to be complex; this impacts the ability of pathologists and clinicians to understand the internal workings of these models.^65^ Similarly, the reproducibility of model output and performance is another challenge that makes their adoption difficult. Having an interactive graphical user interface is essential in AI systems, but such interfaces are either not available entirely or do not provide output visualizations for the intermediate layers of the model. All these challenges reduce users’ trust in AI systems, especially in the case of DP, where decisions directly impact diagnosis and clinical outcomes.^111^

## Conclusion

The integration of multimodal oncology data using advanced computational methods represents a pivotal advancement in the realm of DP and CPATH. The advancements in AI and ML models have enabled a more nuanced understanding of cancer’s multifaceted nature. By synergistically combining clinical, radiological, pathology, and molecular data, researchers and clinicians can gain unprecedented insights into the complex dynamics of cancer, leading to more effective and personalized therapeutic strategies. However, there are multiple associated challenges to multimodal learning. Looking forward, DP stands on the brink of a new era where continuous innovation and interdisciplinary collaboration are key to harnessing the full potential of multimodal data integration. This will undoubtedly be crucial in advancing our understanding of cancer and opening new avenues for patient care and research.
